# Nodavirus Colonizes and Replicates in the Testis of Gilthead Seabream and European Sea Bass Modulating Its Immune and Reproductive Functions

**DOI:** 10.1371/journal.pone.0145131

**Published:** 2015-12-21

**Authors:** Yulema Valero, Marta Arizcun, M. Ángeles Esteban, Isabel Bandín, José G. Olveira, Sonal Patel, Alberto Cuesta, Elena Chaves-Pozo

**Affiliations:** 1 Centro Oceanográfico de Murcia, Instituto Español de Oceanografía (IEO), Carretera de la Azohía s/n. Puerto de Mazarrón, Murcia, Spain; 2 Department of Cell Biology and Histology, Faculty of Biology, Regional Campus of International Excellence "Campus Mare Nostrum", University of Murcia, Murcia, Spain; 3 Unidad de Ictiopatología-Patología Viral, Departamento de Microbiología y Parasitología, Instituto de Acuicultura, Universidad de Santiago de Compostela, Campus Vida, Santiago de Compostela, Spain; 4 Institute of Marine Research, Bergen, Norway; National Cheng Kung University, TAIWAN

## Abstract

Viruses are threatening pathogens for fish aquaculture. Some of them are transmitted through gonad fluids or gametes as occurs with nervous necrosis virus (NNV). In order to be transmitted through the gonad, the virus should colonize and replicate inside some cell types of this tissue and avoid the subsequent immune response locally. However, whether NNV colonizes the gonad, the cell types that are infected, and how the immune response in the gonad is regulated has never been studied. We have demonstrated for the first time the presence and localization of NNV into the testis after an experimental infection in the European sea bass (*Dicentrarchus labrax*), and in the gilthead seabream (*Sparus aurata*), a very susceptible and an asymptomatic host fish species, respectively. Thus, we localized in the testis viral RNA in both species using *in situ* PCR and viral proteins in gilthead seabream by immunohistochemistry, suggesting that males might also transmit the virus. In addition, we were able to isolate infective particles from the testis of both species demonstrating that NNV colonizes and replicates into the testis of both species. Blood contamination of the tissues sampled was discarded by completely fish bleeding, furthermore the *in situ* PCR and immunocytochemistry techniques never showed staining in blood vessels or cells. Moreover, we also determined how the immune and reproductive functions are affected comparing the effects in the testis with those found in the brain, the main target tissue of the virus. Interestingly, NNV triggered the immune response in the European sea bass but not in the gilthead seabream testis. Regarding reproductive functions, NNV infection alters 17β-estradiol and 11-ketotestosterone production and the potential sensitivity of brain and testis to these hormones, whereas there is no disruption of testicular functions according to several reproductive parameters. Moreover, we have also studied the NNV infection of the testis *in vitro* to assess local responses. Our *in vitro* results show that the changes observed on the expression of immune and reproductive genes in the testis of both species are different to those observed upon *in vivo* infections in most of the cases.

## Introduction

Viruses and viral diseases have become one of the unsolved problems in modern aquaculture (since there are no effective preventive measures available to control them) resulting in major economic loses. Amongst the most threatening viruses is nervous necrosis virus (NNV; *Nodaviridae* family, *Betanodavirus* genus), causative agent of the viral encephalopathy and retinopathy (VER), a disease that alters the brain and retina structure and function [1] and provokes mortality rates up to 100% in more than 50 fish species [1, 2]. NNV is a small, naked icosahedral virus, composed of 2 positive single stranded RNA fragments, RNA1 and RNA2, which are capped but not polyadenylated [3]. The capsid is composed of multiple units of a single protein, the coat protein (CP) [4] coded by the RNA2 [3, 5] and involved in host specificity. It has been recently described that each units of the capsid protein (CP) shows three major domains: the N-terminal arm, the shell domain (S-domain) and the protrusion domain (P-domain) formed by three-fold trimeric protrusions with hypervariable surface regions that contribute to host binding and specificity [6]. The RNA1 encodes the viral RNA-dependent RNA polymerase (RdRp). In addition, a sub-genomic RNA transcript, called RNA3, is originated from the 3’ terminus of the RNA1. The RNA3 of betanodavirus has been considered to have a single open reading frame encoding for protein B2 [7]. B2 is important for high-level accumulation of viral RNA1 in the cell and could efficiently antagonize host siRNA silencing [7–9]. Interestingly, B2 is only detected when the virus is actively replicating, but not in persistent infections [10].

Moreover, in the Mediterranean aquaculture, European sea bass (*Dicentrarchus labrax*) is one of the most susceptible species, especially at larvae and juvenile stages, resulting in 100% mortalities at these stages [11, 12]. On the other hand, gilthead seabream (*Sparus aurata*) is less susceptible species than European sea bass and a carrier of the infection for most of the NNV strains [13]. However, this species suffered mortalities when infected with some NNV reassortant strains [14].

Several studies about the transmission mechanisms of the virus point to both horizontal and vertical transmission routes [15–19]. Thus, although adult specimens do not suffer the disease, NNV has been detected in broodstocks of different fish species by PCR and ELISA techniques [1, 20]. The infection of the gonad by pathogens is the initial step to promote horizontal transmission through gonadal fluids and/or vertical transmission through infected gametes [19, 21]. In all vertebrates, the gonad is considered an immunologically privileged site, as also the brain and retina, due to the fact that in those tissues, the immune response proceeds in a different manner in order to avoid cell damage [22, 23]. These physiological characteristics of the gonad are used by a certain number of viral pathogens to evade the immune system, replicate and be transmitted to other specimens. In fish, the immune functions inside the reproductive organs and its implication on pathogen dissemination through the gonad have recently been documented. In rainbow trout (*Oncorhynchus mykiss*), viral haemorrhagic septicaemia virus (VHSV), a *Rhabdovirus*, and infectious pancreatic necrosis virus (IPNV), an *Aquabirnavirus*, can be transmitted through the ovary in two different ways. In fact, some studies have detected infective particles of VHSV in the ovary and ovarian fluids, allowing horizontal and/or vertical transmission through fluids [24], while infective particles of IPNV has only been detected in homogenates of oocytes, being transmitted in a *sensu stricto* vertical way [25]. Interestingly, once they reach the ovary, their replication cycles are completely different. While VHSV is able to actively transcribe and translate its genes and increase the virus load in the tissue, the mRNA levels of IPNV remain undetectable, but infective particles of the virus can be isolated from the ovary by cell culture [26]. In addition, trout leucocytes present into the gonads showed altered immune response (including leucocyte markers, antigen presentation, interferon response, chemokines or cytokines) allowing VHSV to cause chronic infections and IPNV to keep latent into the tissue [26]. Strikingly, there are no studies focused on NNV and its ability to colonize the gonad, even when NNV has demonstrated vertical transmission [15]. In the other hand, sex steroid hormones, regulated by the brain-pituitary-gonadal axis [27, 28] modulate the immune response in vertebrates; including fish (see reviews [29, 30]). As a consequence, some pathogens modify the sex steroid hormone levels of the host when they spread an infection [31, 32].

Taking all this into consideration, in this work we have studied whether NNV colonized the testis in European sea bass and gilthead seabream, a very susceptible and asymptomatic host fish species, respectively. Moreover, we will analyse how the immune response and the production of reproductive hormones, 17β-estradiol (E_2_) and 11-ketotestosterone (11KT) in the testis are modified upon NNV infection. In addition, we determined whether the sensitivity of brain and testis to these reproductive hormones are modified by NNV infection. Furthermore and with the aim to elucidate which of the alterations observed in the testis might be due to NNV localization in this tissue with no interference of other systemic factors or tissue alterations, an *in vitro* challenge of the testis of both species with poly I:C or NNV was performed for 24 hours.

## Materials and Methods

### Animals

Healthy specimens of European sea bass (*Dicentrarchus labrax* L.) and gilthead seabream (*Sparus aurata* L.) were bred and kept at the *Centro Oceanográfico de Murcia* (IEO, Mazarrón, Murcia). The European sea bass larvae were bred at warm water temperature (around 20°C) obtaining a high proportion of males in the population [33]. The fish from juveniles to adults were kept in 14 m^3^ tanks with the natural water temperature, a flow-through circuit, a suitable aeration and filtration system and a natural photoperiod. Fish were fed daily with 1% biomass of a commercial pellet diet (Skretting). The environmental parameters and food intake were recorded daily. Specimens of European sea bass (n = 50) or gilthead seabream (n = 50) of the same age with a mean body weight (bw) of 125 ± 25 and 305 ± 77 g respectively, were transported to the University of Murcia (Spain) aquaria in order to perform *in vivo* infections (see below). Fish were randomly divided into two tanks, kept in 450–500 L running seawater (28 ‰ salinity) aquaria at 25°C and with a 12 h light: 12 h dark photoperiod and acclimatised for 15 days prior to the experiments. Some other specimens with a bw of 509 ± 38 g and 530 ± 148 g, respectively, were used for *in vitro* experiments (see below). Before sampling, all specimens were anesthetized with 40 μl/l of clove oil, completely bled and immediately decapitated and weighed. The experiments described comply with the Guidelines of the European Union Council (2010/63/UE). The protocol was approved by the Committee on the Ethics of Animal Experiments of the *Instituto Español de Oceanografía* (IEO) (Permit Number: 2010/02) and of the University of Murcia (Permit Number: A13150104).

### Nodavirus stock

NNV (strain 411/96, genotype RGNNV) was propagated in the SSN-1 [12]. The SSN-1 cells were grown at 25°C in Leibovitz's L15-medium (Gibco) supplemented with 10% fetal bovine serum (FBS; Gibco), 2 mM L-glutamine (Gibco), 100 IU/ml penicillin (Gibco), 100 μg/ml streptomycin (Gibco) and 50 μg/ml gentamicin (Gibco) using Falcon Primaria cell culture flasks (Becton Dickinson). Inoculated cells were incubated at 25°C until the cytopathic effect (CPE) was extensive. Supernatants were harvested and centrifuged to eliminate cell debris. Virus stock was titrated in 96-well plates and expressed as the viral dilution infecting 50% of the cell cultures (TCID_50_), following the methodology described by Reed and Müench [34], before use in the experiments.

### Testis culture and in vitro treatments

Specimens of European sea bass (n = 6) or gilthead seabream (n = 6) males were completely bled and the testis removed without taking the afferent and efferent blood vessels that are located in the mesentery (dorsally) connecting the testis to the body wall. The testis were weighed and chopped in 1 mm^2^ fragments to culture them in flat-bottomed 96-well microtiter plates (Nunc) with sL-15 culture medium [Leibovitz’s L15-medium supplemented with 2 mM glutamine, 100 u.i./ml penicillin, 100 μg/ml streptomycin, 2 μg/ml fungizone (Life Technologies), 2% FBS serum and 0.35% of NaCl] alone (control) or with NNV (10^7^ TCID_50_/ml) or polyinosinic-polycytidylic acid (poly I:C; a synthetic analog of double-stranded RNA poly I:C) (62.5 μg/ml; Sigma) for 24 hours at 25°C. After incubation, fragments of tissue were washed with 0.01 M phosphate buffered saline (PBS) and processed for gene analysis as described below.

### In vivo infection

The infection was performed either by injecting with a single intramuscular injection of 100 μl of SSN-1 culture medium (mock-infected) or with culture medium containing 10^6^ TCID_50_/fish of NNV since this route of infection has been proven to be the most effective [35]. Mortality was also recorded through the experiment. Fish (n = 5 fish/group and time) were sampled 1, 7 or 15 days after the viral infection and serum, testis and brain were removed. Testis were sampled without taking the afferent and efferent blood vessels that are located in the mesentery (dorsally) connecting the testis to the body wall. Blood samples were obtained from the caudal peduncle and, after clotting; serum samples were collected by centrifugation at 10,000 g for 1 min at 4°C, and immediately frozen and stored at -80°C until use. The testis were weighed and fragments of testis and brain tissues were either immediately frozen in TRIzol Reagent (Life Technologies) and stored at -80°C for later RNA isolation or fixed in 4% paraformaldehyde in PBS for 24 h at 4°C for light microscopy examination as described below.

### Light microscopy and immunocytochemistry

Testis fragments from the *in vivo* experiment (n = 4–5 fish/group and time) fixed in 4% paraformaldehyde in PBS for 24 h at 4°C were embedded in paraffin (Paraplast Plus; Sherwood Medical) and sectioned at 5 μm. After dewaxing and rehydration, some sections were stained with haematoxylin–eosin in order to determine the changes in the morphology of the organs through the infection. Other sections were subjected to a direct immmunocytochemical method using two antibodies specific to: (i) the NNV capsid protein (anti-CP, Ø233 antibody) or (ii) the NNV B2 protein (anti-B2, Ø6073 antibody) at the optimal dilution of 1:500 as previously described [10]. In brief, the sections were incubated at 60°C for 30 min, dewaxed in xylene, rehydrated in a series of ethanol baths and washed in running water. Prior to staining the tissue sections were autoclaved for 15 min in 0.01 mM citric acid (pH 6.0) for antigen retrieval. To prevent non-specific antibody binding, sections were blocked by using 5% bovine serum albumin (BSA; Sigma) in Tris buffered saline (TBS; Merck, 0,05 M, pH 7.6) for 20 min. The primary anti-B2 (Ø6073 antibody) or anti-capsid (Ø233 antibody) sera were diluted in TBS containing 2.5% BSA and incubated for 30 min at 37°C, and washed for 5 min with TBS. The Vectostain^®^ universal ABC-AP kit (Vector Laboratories), which provides both the secondary antibody (biotinylated anti-mouse/rabbit immunoglobulin) and avidin-biotin alkaline phosphatase (ABC-AP) was used. After TBS wash, the sections were incubated for 5 min with DAKO Fuchsin Substrate- and Cromogen system (Dako), followed by washing in running tap water before counterstaining with Shandon’s haematoxylin and mounting in aqueous mounting medium (Aquatex, BDH laboratory). The specificity of the reaction was determined by using sections of tissue from control fish and by omitting the primary antibody on section of tissue from infected fish.

### Localization of gene expression by in situ PCR

Testis sections were used to perform *in situ* PCR (isPCR) analysis using a modified protocol previously described [36]. Sections were dewaxed in xylene for 5 min, dried in 100% ethanol, and air-dried for 5 min. Protease digestion was performed with 2 mg/ml of proteinase K (Invitrogen) for 5 min at room temperature and washed in DEPC–treated water for 1 min. After that, sections were dried with 100% ethanol and air-dried for 1 minute. The sections were treated with DNAse I (300 u/ml; Biotools) for 20 min at room temperature to remove any genomic DNA traces that might interfere with the PCR reactions, washed in DEPC-treated water for 1 min, dried with 100% ethanol, and air-dried for 1 minute. Retrotranscription and amplification reactions were performed using the primers for the gene coding for CP (Table 1) and with MyTaq One-Step RT-PCR kit (Bioline) following the manufacturer’s instructions. All sections were incubated with a total volume of 50 μl of 2% BSA, 50 mM MgCl_2_, 1 mM digoxigenin-11-dUTP alkali-stable buffer 5x, 10 u/μl Ribosafe RNAse inhibitor and 5 μl of Reverse Transcriptase for 20 min at 45°C and 1 min at 95°C. Amplification was carried out by running, 25 cycles for 10 s at 95°C, 10 s at 60°C and 30 s at 72°C; and a final step of 10 min at 72°C. Afterwards, all sections were washed with 2% BSA in 1x SSC [15 mM sodium citrate dihydrate and 0.15 M NaCl] during 10 min at 52°C. The digoxigenin-11-dUTP incorporated in the PCR products was detected by means of a direct immuncytochemistry method using a specific antiserum anti-digoxigenin-HPR (Roche) in 0.1 M Tri-HCl with 0.1 M NaCl at the optimal dilution of 1:100 for 60 min at room temperature. The peroxidase activity was revealed by incubation with 0.05% 3,3’-diaminobenzidine tetrahydrochloride (DAB) in 0.01 M Tris-HCl with 0.1 M NaCl and 0.05% H_2_O_2_ at room temperature for 5 min. Tissue from non-infected fish was used as negative controls. In order to determine the ability of the amplified products to diffuse into the aqueous phase, liquid phase was collected after performing the *in situ* RT-PCR reactions, run in 1% agarose gel for electrophoresis (Bioline) with 0.5 μg/ml of ethidium bromide (Sigma) and visualized under UV light.

**Table 1:** Information about the studied genes, primer sequences and application used in this work.

	Protein	RNA molecule or gene abbreviation	Accession number	Sequence (5’-3’)		Tm	Use	Tissue
NNV	Capsid protein	*cp*	D38636	F	AAATTGCACACCACCTGTGA	60°C	TaqMan real-time PCR	Testis
				R	ACCCAGAATGGAATGTCAGC			
				Probe	6FAM-ACTGCACGTGTGGTCCAGTA-MGB			
				F	CGTGTCAGTCATGTGTCGCT	60°C	SYBR real-time PCR / *in situ* PCR	Testis / E-11
				R	CGAGTCAACACGGGTGAAGA			
				F2	CGTGTCAGTCATGTGTCGCT	58°C	Conventional PCR	Brain
				R3	CGAGTCAACACGGGTGAAGA			
	RNA-dependent RNA polymerase	*rdrp*	AF319555	F	GAGGGTGCGATTGCTATTGT	60°C	TaqMan real-time PCR	Testis
				R	ACTGGCACCCAATTAAGCAC			
				Probe	6FAM-CGCTTGAAGGCCTATACACG-MGB			
Gilthead seabream	Tumor necrosis factor alpha	*tnfa*	AJ413189	F	TCGTTCAGAGTCTCCTGCAG	60°C	SYBR real-time PCR	Testis Brain
				R	TCGCGCTACTCAGAGTCCATG			
	Interleukin 6	*il6*	AM749958	F	AGGCAGGAGTTTGAAGCTGA	60°C	SYBR real-time PCR	Testis Brain
				R	ATGCTGAAGTTGGTGGAAGG			
	Interleukin 1 beta	*il1b*	AJ277166	F	GGGCTGAACAACAGCACTCTC	60°C	SYBR real-time PCR	Testis Brain
				R	TTAACACTCTCCACCCTCCA			
	T cell receptor beta chain	*tcrb*	AM261210	F	AAGTGCATTGCCAGCTTCTT	60°C	SYBR real-time PCR	Testis Brain
				R	TTGGCGGTCTGACTTCTCTT			
	Immunoglobulin M heavy chain	*igmh*	AM493677	F	CAGCCTCGAGAAGTGGAAAC	60°C	SYBR real-time PCR	Testis Brain
				R	GAGGTTGACCAGGTTGGTGT			
	Double sex-and mab3- related transcription factor 1	*dmrt1*	AM493678	F	GATGGACAATCCCTGACACC	60°C	SYBR real-time PCR	Testis
				R	GGGTAGCGTGAAGGTTGGTA			
	Gonadal aromatase	*cyp19a1a*	AF399824	F	CACCATGGATCTGATCTCTGCCTGT	60°C	SYBR real-time PCR	Testis
				R	GAGCGTTTGCCAGCTGCCTC			
	Steroid 11-β-hydroxylase	*cyp11b1*	FP332145	F	GCTATCTTTGGACCCCATCA	60°C	SYBR real-time PCR	Testis
				R	CTTGACTGTGCCTTTCAGCA			
	Estrogen receptor α	*era*	AF136979	F	GCTTGCCGTCTTAGGAAGTG	60°C	SYBR real-time PCR	Testis Brain
				R	TGCTGCTGATGTGTTTCCTC			
	Estrogen receptor β1	*erb1*	AF136980	F	CAGCTCCAGAAGGTGGACTC	58°C	PCR	Testis Brain
				R	GGATTGGCATAGCTGAAAT			
	Estrogen receptor β2	*erb2*	AJ580050	F	TGATGATGTCACTCACCAACC	58°C	PCR	Testis Brain
				R	TTCAGCTCACGAAACCGA			
	Elongation factor 1α	*ef1a*	AF184170	F	CTGTCAAGGAAATCCGTCGT	60°C	SYBR real-time PCR	Testis Brain
				R	TGACCTGAGCGTTGAAGTTG			
	β-Actin	*actb*	X89920	F	ATCGTGGGGCGCCCCAGGCACC	55°C	PCR	Testis Brain
				R	CTCCTTAATGTCACGCACGATTTC			
European sea bass	Tumor necrosis factor alpha	*tnfa*	DQ200910	F	CGAGGGCAAGACTTTCTTTG	60°C	SYBR real-time PCR	Testis Brain
				R	GCACTGCCTGTTCAGCTACA			
	Interleukin 6	*il6*	AM490062	F	ACTTCCAAAACATGCCCTGA	60°C	SYBR real-time PCR	Testis Brain
				R	CCGCTGGTCAGTCTAAGGAG			
	Interleukin 1 beta	*il1b*	AJ269472	F	CAGGACTCCGGTTTGAACAT	60°C	SYBR real-time PCR	Testis Brain
				R	GTCCATTCAAAAGGGGACAA			
	T cell receptor beta chain	*tcrb*	FN687461	F	GACGGACGAAGCTGCCCA	60°C	SYBR real-time PCR	Testis Brain
				R	TGGCAGCCTGTGTGATCTTCA			
	Immunoglobulin M heavy chain	*igmh*	FN908858	F	AGGACAGGACTGCTGCTGTT	60°C	SYBR real-time PCR	Testis Brain
				R	CACCTGCTGTCTGCTGTTGT			
	Gonadal aromatase	*cyp19a1*	AJ298290AJ311177	F	CTGGAGCCACACAGACAAGA	60°C	SYBR real-time PCR	Testis
				R	AACTGAGGCCCTGCTGAGTA			
	Neural aromatase	*cyp19a2*	AY138522	F	CATGTTCTGAGGAGCGTTCA	60°C	SYBR real-time PCR	Testis
				R	AAGGGAGTCCACATGTCCTG			
	Steroid 11-β-hydroxylase	*cyp11b1*	AF449173	F	CCCATCTACAGGGAGCATGT	60°C	SYBR real-time PCR	Testis
				R	GGAAGACTCCTTTGCTGTGC			
	Estrogen receptor β1	*erb1*	AJ489523	F	GGGTGAGAGAGCTCAAGCTC	60°C	SYBR real-time PCR	Testis Brain
				R	AAGCTAAGGCCGGTTTTGGC			
	Estrogen receptor β2	*erb2*	AJ489524	F	AGTGGGCATGATGAAGTGCG	60°C	SYBR real-time PCR	Testis Brain
				R	TGCACGTGGTTCACCTGAGG			
	Elongation factor 1α	*ef1a*	FM019753	F	CGTTGGCTTCAACATCAAGA	60°C	SYBR real-time PCR	Testis Brain
				R	GAAGTTGTCTGCTCCCTTGG			
	β-Actin	*actb*	AJ493428	F	ATCGTGGGGCGCCCCAGGCACC	55°C	PCR	Brain
				R	CTCCTTAATGTCACGCACGATTTC			

### Isolation of NNV infective particles from the testis

After 15 days of infection, testis fragments from mock- or NNV-infected fish were homogenized in 1 ml of 0.01 M PBS. E-11 cells [37], derived from the SSN-1, cultured in L-15 culture medium supplemented with antibiotics and 2% FBS were inoculated with the testis samples and incubated at 25°C. Infected monolayers were examined daily for the presence of cytopathic effect (CPE). Those cultures showing CPE were further processed to isolate the total RNA and confirm the identity of NNV by real-time PCR as described below.

### cDNA synthesis

Total RNA was extracted from testis and brain fragments from both, the *in vivo* (n = 4–5 fish/group and time) and the *in vitro* (n = 6 fish/group) experiments, with TRIzol Reagent (Life Technologies) following the manufacturer’s instructions, and quantified with a spectrophotometer (Cecil Instruments Ltd). In the cell culture experiments total RNA was extracted using RNeasy Mini kit (Qiagen) following the manufacturer´s instructions.

Isolated RNA was DNase I treated (amplification grade, 1 unit/μg RNA, Life Technologies) and the SuperScript III RNase H−Reverse Transcriptase (Life Technologies) was used to synthesize first strand cDNA with 1 μl of random primers (0.25 μg/μl; Life Technologies) from 1 μg of total RNA, at 50°C for 60 min.

### Confirmation of NNV gene expression

With the aim of determining the levels of transcription of RNA-dependent RNA polymerase (*rdrp*) and capsid protein (*cp*) genes of NNV in the testis and brain of *in vivo* infected gilthead seabream and European sea bass specimens, real-time PCR with TaqMan probe was performed with an ABI PRISM 7500 instrument (Applied Biosystems) using TaqMan® Gene Expression Master Mix (Applied Biosystems). The primers and TaqMan fluorogenic probes are detailed in Table 1. Reaction mixtures were incubated for 2 min at 50°C and subsequently 10 min at 95°C, followed by 40 cycles of 15 s at 95°C, 1 min at 60°C, and finally 15 s at 95°C, 1 min 60°C and 15 s at 95°C to finish the reaction. To confirm the results, conventional PCR using the standard F2 and R3 primers for NNV T4 region was also applied to brain of mock-infected and NNV infected sea bass specimens as described elsewhere [38].

In order to identify the presence of NNV in the E-11 cell line inoculated with testis homogenates from control and *in vivo* infected fish at day 15 post-infection, real-time PCR reactions were carried out in a final volume of 50 μl, containing 200 nM of each primer coding for the *cp* gene of NNV (Table 1), as previously described [39], and 2 μl of cDNA template in iQ™ SYBR® Green Supermix (Bio-Rad). Following an initial 15 min denaturation/activation step at 95°C, the mixture was subjected to 45 cycles of amplification (denaturation for 15 s at 95°C, annealing and extension for 15 s at 60°C) in a CFX96™ Real-time PCR detection system (BioRad).

### Evaluation of immune- and reproductive-related genes expression

The gilthead seabream and European sea bass genes coding for: (i) pro-inflammatory cytokines such as the tumor necrosis factor alpha (*tnfa*) and the interleukin 6 (*il6*) and 1 beta (*il1b*); (ii) specific cellular immune response markers as the beta chain of the T cell receptor (*tcrb*) and the heavy chain of the immunoglobulin M (*igmh*); (iii) the sex specific gene double sex-and mab3-related transcription factor 1 (*dmrt1*); (iv) esteroidogenic enzymes such as gonadal aromatase (*cyp19a1a* of gilthead seabream or *cyp19a1* of European sea bass), neural aromatase (*cyp19a2*) and steroid 11-β-hydroxylase (*cyp11b1*); and (v) the estrogen receptors as the estrogen receptor α (*era*) of gilthead seabream, and the European sea bass estrogen receptor β1 (*erb1*) and β2 (*erb2*) were analysed in the testis and brain by real-time PCR using an ABI PRISM 7500 instrument and SYBR® Green PCR Core Reagents (Applied Biosystems) as previously described [26]. The specific primers are shown in Table 1. For each sample, gene expression was normalised by its *ef1a* content presented as 2^-ΔCt^, where ΔCt is determined by subtracting the elongation factor 1 alpha (*ef1a*) Ct value from the target Ct. Before the experiments, the specificity of each primer pair was studied using positive and negative samples. A melting curve analysis of the amplified products validated the primer for specificity. Negative controls with no template were always included in the reactions.

The *erb1* and *erb2* genes of gilthead seabream were analysed by semi-quantitative PCR performed with a Flexcycler (Analitikjena). Reaction mixtures were incubated for 2 min at 94°C, followed by 35 cycles of 45 s at 94°C, 45 s at the specific annealing temperature for each gene (see Table 1), 1 min at 72°C, and finally 10 min at 72°C. For visualizing and comparing the groups, the PCR products were run on a 2% agarose gel. As internal control, the expression of β-actin coding gene (*actb*) was used.

### Serum sex hormone levels

Serum levels of 17β-estradiol (E_2_) and 11-ketotestosterone (11KT) were quantified by ELISA following the method previously described in European sea bass [40] and adapted to gilthead seabream [41]. Steroids were extracted from 10 or 20 μl individual serum (n = 5 fish/group and time) from sea bass or seabream, respectively, in 1.3 ml of methanol (Panreac). Then, methanol was evaporated at 37°C and the steroids were resuspended in 400 μl of reaction buffer [0.1 M phosphate buffer with 1 mM EDTA (Sigma), 0.4 M NaCl (Sigma), 1.5 mM NaN_3_ (Sigma) and 0.1% BSA]. 50 μl of extracted sample (1.25 or 2.5 μl of serum per reaction, respectively) were used for each ELISA reaction. The standard, mouse anti-rabbit IgG monoclonal antibody (mAb), and specific anti-steroid antibodies and enzymatic tracers (steroid acetylcholinesterase conjugates) were obtained from Cayman Chemical while the microtiter plates (MaxiSorp) were purchased from Nunc. A standard curve from 6.13 x 10^−4^ to 5 ng/ml (0.03–250 pg/well), a blank and a non-specific binding control (negative control) was established in all the assays. Standards and extracted serum samples were run in duplicate. The lower limit of detection for European sea bass assays was 24.41 pg/ml and for gilthead seabream assays was 12.21 pg/ml. The intra-assay coefficients of variation (calculated from sample duplicates) were 9.3 ± 4.3% for E_2_ and 9.1 ± 3.8% for 11KT assays for serum. Details on cross-reactivity for specific antibodies were provided by the supplier (0.01% of anti-11KT reacts with T; and 0.1% of anti-E_2_ reacts with T; no cross-reaction between 11KT and E_2_ was described).

### Calculations and statistical analysis

The gonads were weighed and the gonadosomatic index (GSI) was calculated as an index of the reproductive stage [100*(MG/MB) (%)], where MG is gonad mass (in grams), and MB is body mass (in grams).

All slides were examined with a Nikon eclipse E600 light microscope. The images were obtained with an Olympus SC30 digital camera (Olympus soft imaging solutions GMBH) and Spot 3.3 software (Diagnostic instruments).

The genetic nomenclature used in this manuscript follows the guidelines of Zebrafish Nomenclature Committee (ZNC) for fish genes and proteins and the HUGO Gene Nomenclature committee for mammalian genes and proteins.

The quantification of gilthead seabream *erb1* and *erb2* gene expression was determined by means of 2% agarose gel densitometrically scanned using an image analysis using the Gel Logic 100 Imaging System (Kodac) and ImageJ 1.44p software (National Institute of Health). The data are showed as the mean value ± standard error to the mean (SEM) of the gene expression relative to *actb* gene expression.

All data were analysed by t-Student test to determine statistical differences between infected and control groups, or one-way ANOVA to denote statistical differences at different points in the infected group (P ≤ 0.05). A non-parametric Kruskal–Wallis test, followed by a multiple comparison test, was used when data did not meet parametric assumptions. Statistical analyses were conducted using SPPS 15.0 application. All data are presented as mean ± SEM. Minimum level of significance was fixed in 0.1 (*P ≤ 0.1; **P ≤ 0.05; ***P ≤ 0.01). Letters denote statistical differences between different time points (P ≤ 0.05).

## Results

**Fig 1:**
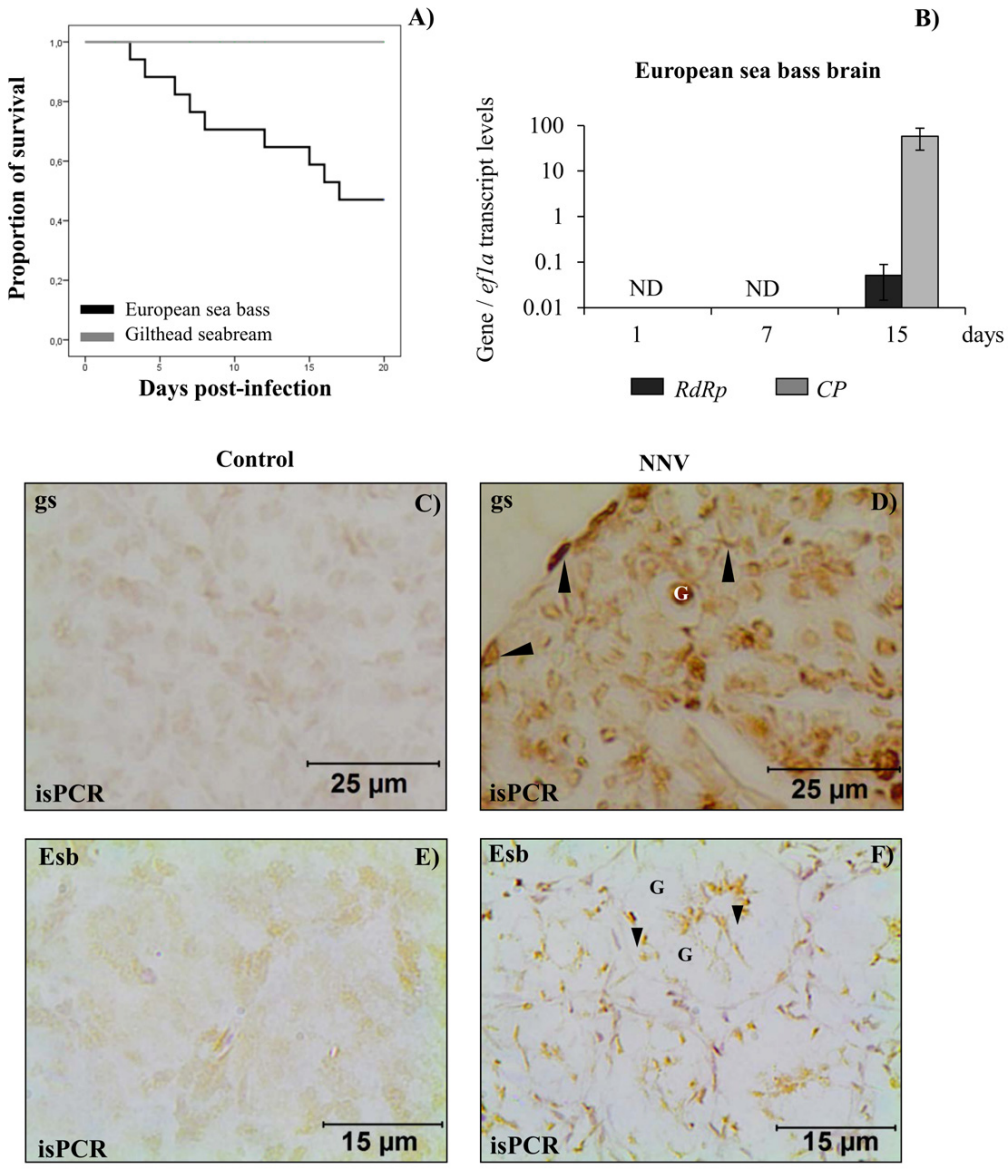
Nodavirus causes mortality and viral RNA is detected in the brain and testis. (A) Kaplan-Meier survival curves showing the proportion of European sea bass (black line) and gilthead seabream (grey line) survivors after intramuscular injection with 10^6^ TCID_50_ nodavirus/fish. (B) Transcript levels of the NNV genes, coding for polymerase (*RdRp*) and capsid (*CP*) proteins, analysed by real-time-PCR with TaqMan fluorogenic probes, in European sea bass brain after 1, 7 and 15 days of *in vivo* infection with NNV. Viral genes expression was never detected in mock-infected samples and is not shown. (C-F) Detection of RNA coding for the CP protein of NNV by *in situ* PCR (isPCR) in the testis of gilthead seabream (C,D) or European sea bass (E,F) after 15 days of *in vivo* infection with NNV. Control group (mock infected; C,E) and NNV group (10^6^ TCID_50_/fish; D,F). Germ cells (G) and somatic cells (arrow head) are labelled in gilthead seabream (D), while in the European sea bass, somatic cells, mainly the Sertoli cells located between germ cells were stained (F). Scale bars = 25 μm (C,D) or 15 μm (E,F).

The experimental infection showed no mortality or disease signs in gilthead seabream specimens, but reached 55% mortality in European sea bass (Fig 1).

### NNV colonizes and replicates in the testis of gilthead seabream and European sea bass

**Fig 2:**
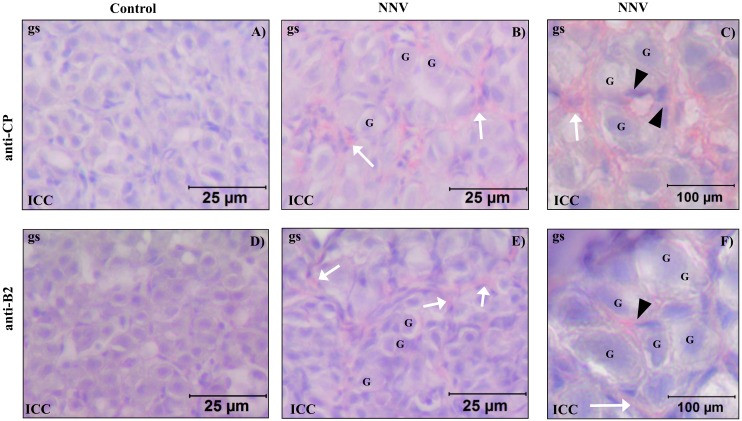
The NNV proteins CP and B2 are detected in the testis of infected gilthead seabream specimens. (A-F) Immunocytochemistry in paraffin embedded sections of gilthead seabream testis of mock infected (Control; A,D) or NNV infected (10^6^ TCID_50_/fish) specimens (NNV; B,C,E,F) at day 15 using the anti-capsid (Ø233 antibody; 1:500; A,B,C) or the anti-B2 (Ø6073 antibody; 1:500; D,E,F) sera. Scale bars = 25 μm (A,B,D,E) and 100 μm (C,F). Infected cells are shown in dark red stain. The interstitial cells (white arrows) are located surrounding the tubules and the Sertoli cells (arrow heads) located inside the tubules and between the unstained germ cells (G).

All the techniques performed to detect NNV were firstly applied to control fish confirming that these fish were free of NNV (Figs 1 and 2 and Figure A in S1 File).

In the testis and brain of gilthead seabream and European sea bass specimens experimentally infected *in vivo* with NNV were determined the transcription levels of the mRNA coding for the two main proteins of the virus, the RdRP and the CP proteins, using a specific probe for each, by TaqMan real-time PCR. Surprisingly, no transcripts of these genes were found in the testis of neither gilthead seabream nor European sea bass, neither in the gilthead seabream brain (data not shown). However, both were detected in the brain of European sea bass after 15 days of infection (Fig 1A).

The *cp* gene in the testis of gilthead seabream and European sea bass analysed by isPCR determined transcripts of this gene in the testicular cells of both species at 15 days post infection (Fig 1B–1E and Table 2). Thus, the *cp* mRNA was found in somatic cells (black arrow head in Fig 1D) and germ cells (G in Fig 1D) in the testis of gilthead seabream (Fig 1C), while in European sea bass testis, the mRNA of the *cp* was localized only in somatic cells (black arrow head in Fig 1E), but not in germ cells (G in Fig 1E). After analysing by electrophoresis the liquid phase of each isPCR reaction, we found no amplicons, confirming that there is no diffusion of PCR products between cells (Figure B in S1 File).

**Table 2:** The number of NNV positive fish/total number of fish analyzed. The analyses were performed in the testis of fish with three different techniques upon 15 days of infection except for the data in bold that correspond to samples obtained upon 28 days of infection.

Detection technique	Gilthead seabream	European sea bass
isPCR	1/5	3/5
isPCR	**4/5**	
Immunocytochemistry	**3/5**	0/5
Virus recovery	2/5	1/5

Conventional light microscopy showed that at 15 days post infection, the testis of both species had normal morphology characteristic of the resting stage of the reproductive cycle, in which the tubules of the testis are only formed by Sertoli cells that enclosed germ cells [42–44]. Interestingly, using specific antibodies against CP (Fig 2A, 2B and 2C) and B2 (Fig 2D, 2E and 2F), both NNV proteins were localized in somatic cells of the testis. Taking into account the morphology of fish testis (For review on fish testicular morphology see [45]), we observed stained Sertoli cells, located inside the tubules and between non stained germ cells (black arrow heads in Fig 2C and 2F), and stained interstitial cells, located around the tubules (white arrows in Fig 2B, 2C, 2E and 2F) of the testis of gilthead seabream after 15 days of NNV infection (Fig 2B, 2C, 2E and 2F and Table 2). However, none of these proteins were found in European sea bass testis (data not shown and Table 2).

**Fig 3:**
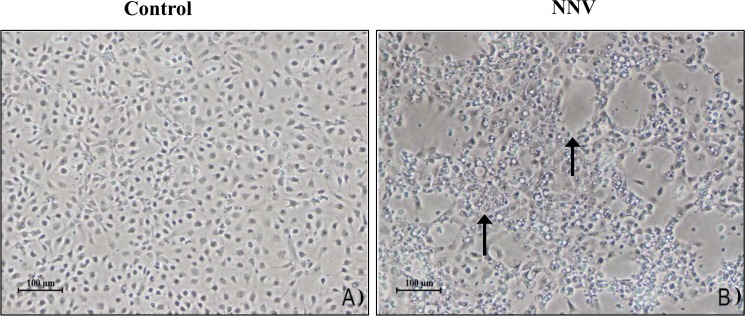
Recovery of infective particles of NNV from testis of gilthead seabream and European sea bass. (A-B) Cytopathic effect (CPE, arrow) in a monolayer of E-11 cells inoculated with testis homogenates from control (A) or infected (B) specimens after 15 days of *in vivo* infection with NNV. Scale bars = 100 μm (A,B).

In order to confirm the presence of infective viral particles of NNV in the testis, the permissive E-11 cell line monolayers were incubated with testis homogenates from infected fish of both species. Although none of the inoculated monolayers developed extensive CPE after 10 days of inoculation, after a blind passage, partial CPE was observed in three cell cultures inoculated with three samples, two from gilthead seabream and one from European sea bass infected testis. The CPE was characterized by partial disintegration of the monolayer and rounded granular cells with vacuoles (Fig 3 and Table 2). The identity of isolates was confirmed by real-time PCR (data not shown and Table 2). These data determine that NNV reached the testis and probably maintained very low expression levels of its proteins.

### NNV triggers immune response in the testis and brain of European sea bass but not in gilthead seabream

**Fig 4:**
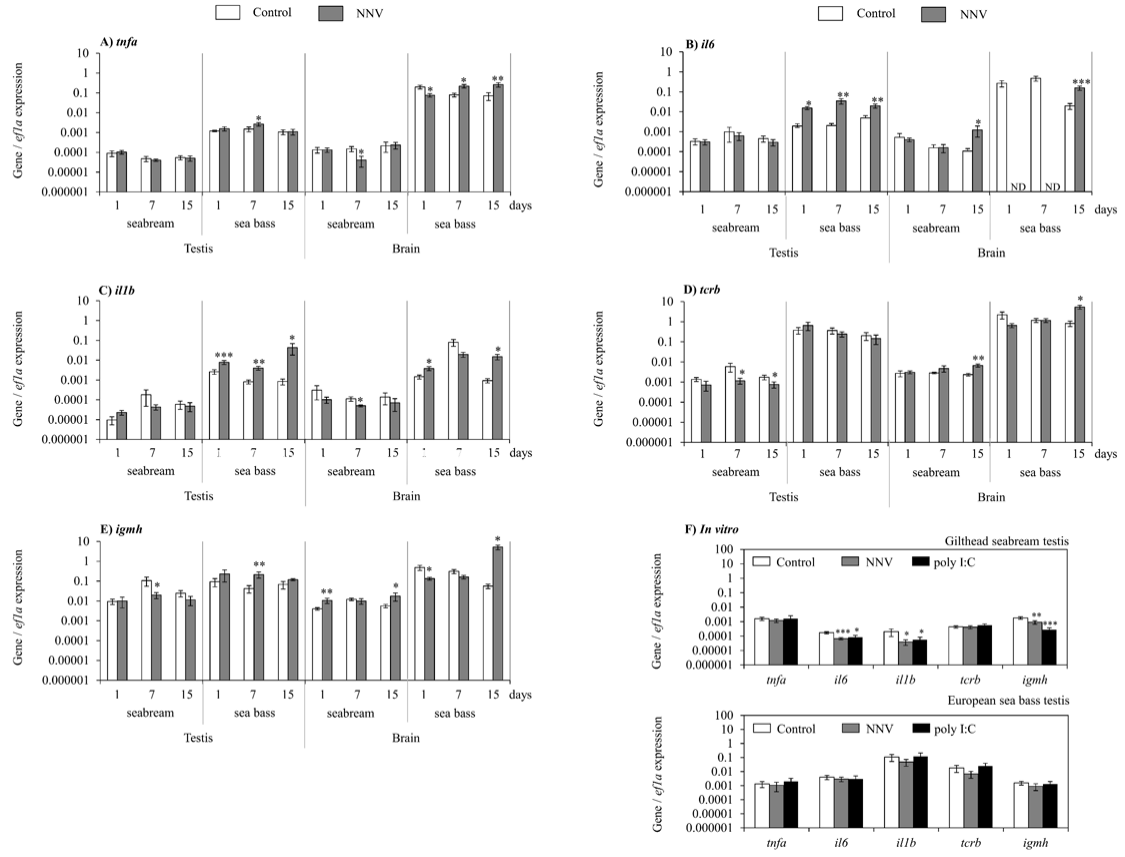
NNV triggers immune responses in the tesis and brain of European sea bass but not in gilthead seabream. (A-E) Transcription of *tnfa* (A), *il6* (B), *il1b* (C), *tcrb* (D) and *igmh* (E) genes in the testis and brain of gilthead seabream and European sea bass, after 1, 7 and 15 days of *in vivo*. (F) Transcription of *tnfa*, *il6*, *il1b*, *tcr* and *igmh* genes after 24 hours of *in vitro* infection with NNV. Data represent the mean ± standard error of the mean (n = 5/group and time). Significance level (P) was fixed at 0.1 (P≤0.1*; P≤0.05**; P≤0.01***). ND, not detected.

Once we knew that the NNV colonized and/or replicated in the testis and brain of gilthead seabream and European sea bass, we studied the pattern of expression of pro-inflammatory cytokines *tnfa*, *il6* and *il1b* and T and B lymphocyte markers (*tcrb* and *igmh*, respectively) genes in both tissues and species upon *in vivo* infection and in the testis upon *in vitro* infection (Fig 4) and found that, in gilthead seabream (Fig 4A–4E), all the cytokine genes were unchanged in the testis (Fig 4A–4E), whilst in the brain, the *tnfa* and *il1b* gene expression was down-regulated after 7 days of infection (Fig 4A and 4C) and the *il6* gene expression was increased after 15 days of infection (Fig 4B). Regarding the lymphocyte markers, the *tcrb* gene was down-regulated from day 7 onwards in the testis and up-regulated at day 15 in the brain (Fig 4D). Similarly, the *igmh* gene expression was down-regulated at day 7 in the testis and up-regulated at day 1 and 15 in the brain (Fig 4E). When the testis was *in vitro* challenged with NNV or poly I:C the expression levels of all these genes were down-regulated (*il6*, *il1b*, *igmh*) or unchanged (*tnfa*, *tcrb*) (Fig 4F).

In contrast, in the European sea bass upon *in vivo* infection (Fig 4A–4E), all the cytokine genes analysed, were up-regulated at least at one time point in the testis (Fig 4A–4E). In the brain, however, the *tnfa* gene expression was down-regulated at day 1 and up-regulated from day 7 onwards (Fig 4A) and the *il6* gene expression was completely blocked at days 1 and 7 and up-regulated at day 15 (Fig 4B). Finally, the *il1b* gene expression was up-regulated after 1 and 15 days of infection (Fig 4C). Regarding the lymphocyte marker genes, the *tcrb* transcription levels were only up-regulated at day 15 in the brain and kept unmodified in the testis (Fig 4D), while the *igmh* transcription levels were up-regulated at day 7 in the testis and down and up-regulated at day 1 and 15 in the brain, respectively (Fig 4E). None of these genes were modified in the testis upon an *in vitro* challenge with NNV or poly I:C (Fig 4F).

### NNV alters steroidogenesis and sex steroid hormones in the testis of gilthead seabream and European sea bass

**Fig 5:**
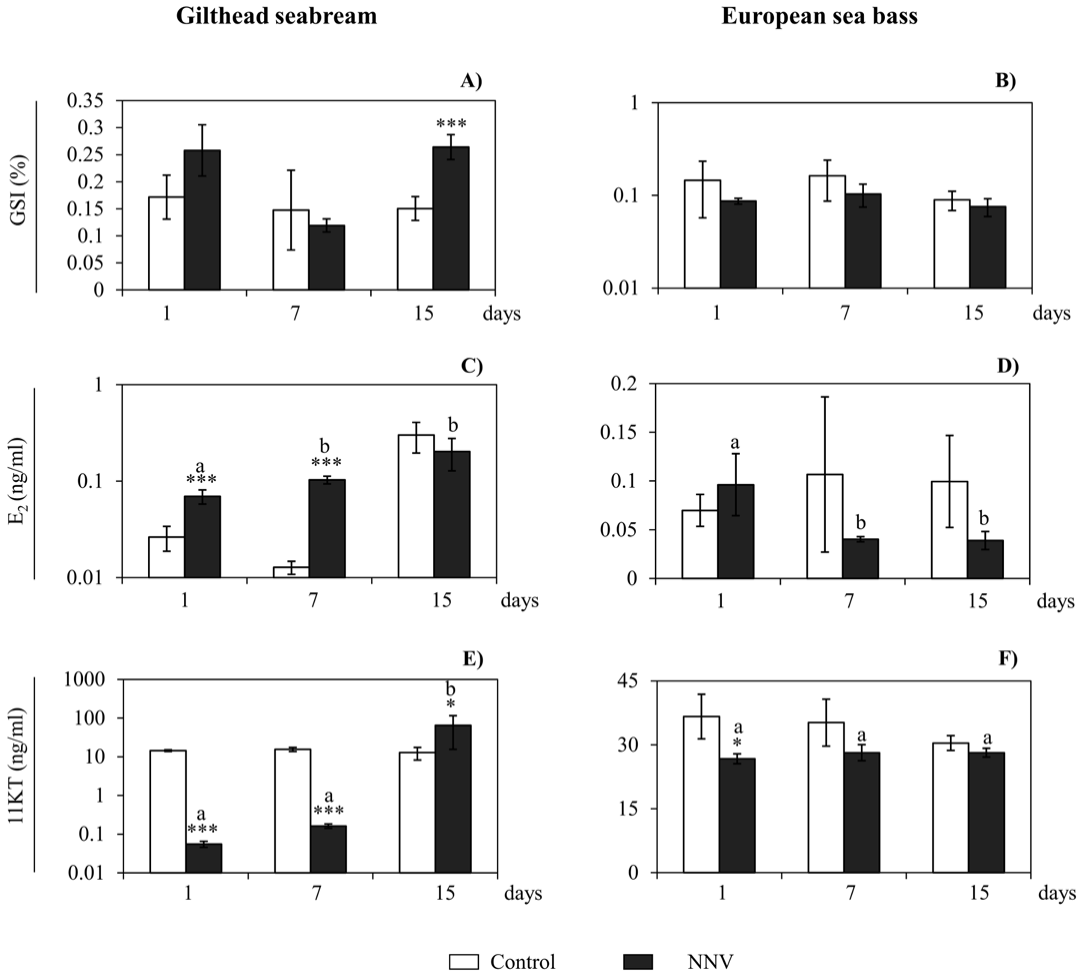
NNV alters sex steroid hormones in gilthead seabream and sea bass. (A-B) Gonadosomatic index **(**GSI) in gilthead seabream (A) and European sea bass (B) after 1, 7 and 15 days of *in vivo* infection with NNV. (C-F) Sex steroid serum levels of 17β-estradiol (E_2_; C,D), and 11-ketotestosterone (11KT; E,F) in gilthead seabream (C,E) and European sea bass (D,F) after 1, 7 and 15 days of *in vivo* infection with NNV. Control group (mock infected) and NNV group (10^6^ TCID_50_/fish). Data represent the mean ± standard error of the mean (n = 5/group and time). Significance level (P) was fixed at 0.1 (P≤0.1*; P≤0.01***). Letters denote statistically significant differences within the infected group over time (P≤0.05).

As an index of the reproductive stage, we analyzed the GSI and the E_2_ and 11KT serum levels (Fig 5) and found that in the gilthead seabream, the GSI was increased after 15 days of NNV infection (Fig 5A), while in the European sea bass no changes were observed (Fig 5B). Regarding the hormonal levels in serum (Fig 5C–5F), in gilthead seabream, NNV induced a high increment in E_2_ serum levels after 1 and 7 days of infection (Fig 5C), whereas in European sea bass E_2_ serum levels were not modified compared to controls (Fig 5D). On the other hand, the 11KT levels in gilthead seabream serum were strongly decreased at day 1 and 7 and increased at day 15 upon infection (Fig 5E), while in European sea bass, 11KT serum level was only decreased at 1 day of infection (Fig 5F). Interestingly as the infection progressed an increment in the serum level of E_2_ and 11KT were observed in gilthead seabream (Fig 5C and 5E) but not in European sea bass, where the E_2_ serum levels decrease throughout the infection period (Fig 5F).

**Fig 6:**
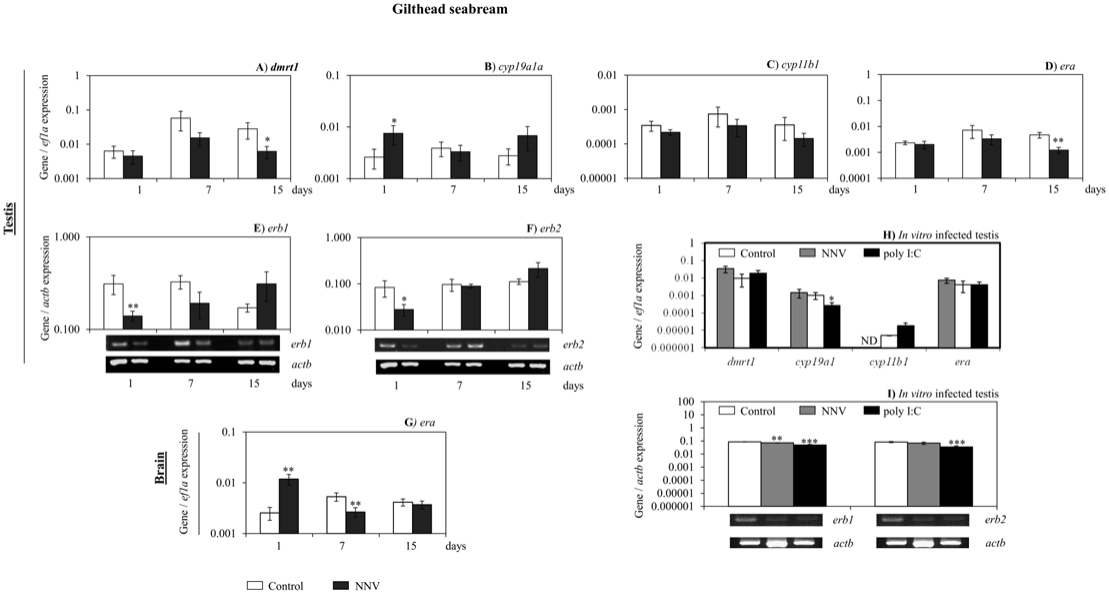
NNV alters the expression of some reproductive and steroidogenic relevant genes in gilthead seabream testis. (A-F) Transcription of *dmrt1* (A), *cyp19a1a* (B), *cyp11b1* (C), *era* (D), *erb1* (E) and *erb2* (F) in the testis of gilthead seabream after 1, 7 and 15 days of *in vivo* infection with NNV. (G)Transcription of *era* in the brain of gilthead seabream after 1, 7 and 15 days of *in vivo* infection with NNV. (H-I) Transcription levels of *dmrt1*, *cyp19a1a*, *cyp11b1*, *era*, *erb1* and *erb2* after 24 hours of *in vitro* infection with NNV. Data represent the mean ± standard error of the mean (n = 5/group and time). Significance level (P) was fixed at 0.1 (P≤0.1*; P≤0.05**; P≤0.01***).

In order to determine whether the NNV infection or the changes in the sex-steroid hormone levels detected in gilthead seabream serum affects the functionality of the testis, we next analysed the expression of some reproduction-related genes in the testis. Firstly, *dmrt1* gene expression, a marker of male function in the gilthead seabream testis [44], was slightly down-regulated at day 15 upon infection (Fig 6A). Secondly, the expression of genes coding for aromatase (*cyp19a1a*) and 11β-hydroxilase (*cyp11b1*), the enzymes involved in E_2_ and 11KT production, respectively; and several E_2_ nuclear receptors (*era*, *erb1* and *erb2*) were differently regulated. The *cyp19a1a* expression was up-regulated at 1 day post infection (Fig 6B), whilst the *cyp11b1* expression was unchanged (Fig 6C). On the other hand, all estrogen nuclear receptor genes were down-regulated at different time points (Fig 6D, 6E and 6F). As the E_2_ regulated the reproductive behaviour of fish through signalling by its receptor in the brain, the target tissue of the NNV, we also analyzed the expression of *era* in this tissue (Fig 6G), and found that *era* transcript levels were increased at day 1 and decreased at day 7 (Fig 6G), whereas *erb1* and *erb2* gene expressions were undetected in the brain of both controls and infected fish samples (data not shown). Interestingly, when the expression pattern of these genes was analysed in the testis upon *in vitro* challenge with NNV or poly I:C, we found that NNV up-regulated the *cyp11b1* and down-regulated the *erb1* gene expression, while poly I:C up-regulated the *cyp11b1* and down-regulated the *cyp19a1a*, *erb1* and *erb2* gene expression.

**Fig 7:**
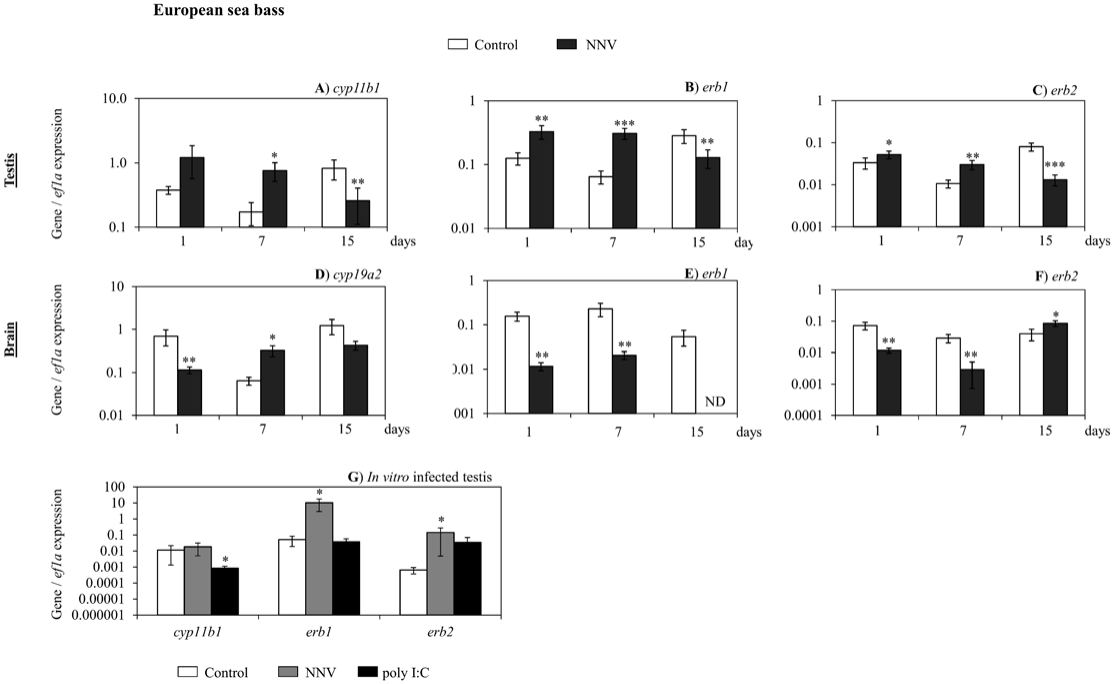
NNV alters the expression of some reproductive and steroidogenic relevant genes in European sea bass testis. (A-C) Transcription of *cyp11b1* (A), *erb1* (B) and *erb2* (C) in the testis of European sea bass after 1, 7 and 15 days of *in vivo* infection with NNV. (D-F) Transcripion of *cyp19a2* (D), *erb1* (E) and *erb2* (F) in the brain of European sea bass after 1, 7 and 15 days of *in vivo* infection with NNV. (G)Transcripion of *cyp11b1*, *erb1* and *erb2* after 24 hours of *in vitro* infection with NNV. Data represent the mean ± standard error of the mean (n = 5/group and time). Significance level (P) was fixed at 0.1 (P≤0.1*; P≤0.05**; P≤0.01***). ND, not detected.

Regarding the European sea bass, we found that the steroidogenic enzymes and hormonal receptor genes analysed were also altered upon *in vivo* infection with NNV in the testis and the brain. Thus, the expression of *cyp11b1* was up-regulated at day 7 and *erb1* and *erb2* genes at day 1 and 7, respectively, while the three genes were down-regulated at day 15 of infection (Fig 7A, 7B and 7C). The *cyp19a1* gene expression was undetectable in both control and infected specimens (data not shown). However, in the brain, nodavirus modify the expression pattern of *cyp19a2*, the neural aromatase, which was down-regulated at day 1 and up-regulated at day 7 post infection (Fig 7D). Otherwise, *erb1* and *erb2* transcription was decreased after 1 and 7 days of infection (Fig 7E and 7F). In addition, NNV infection completely blocked the expression of the *erb1* gene after 15 days of infection, when *erb2* gene expression was up-regulated (Fig 7E and 7F).

Finally, in the testis of European sea bass challenged *in vitro* with NNV and poly I:C (Fig 7G), the *cyp19a1* gene expression was undetectable even in the control fish (data not shown), whereas the expression of *cyp11b1* gene was down-regulated upon poly I:C exposure. Interestingly, the transcription of the genes coding for both estrogen receptors, *erb1* and *erb2*, was up-regulated upon NNV challenge.

## Discussion

NNV is a single stranded RNA virus which causes VER disease and provokes high mortality rates in several Mediterranean fish species [1]. European sea bass is one of the most susceptible species to this disease and undergoes high mortalities at larvae and juvenile stages [11, 12]. However, other species such as gilthead seabream have been considered to be an asymptomatic carrier, although recent outbreaks of VER disease resulting in high mortalities have threatened this species too [14, 46]. NNV is a known vertically transmitted pathogen [1, 18]. The knowledge about the ability of NNV to colonize and evade the immune response in the gonad represents the initial step to understand how pathogens are vertically transmitted to the progeny and also potentially improve and develop new strategies to prevent NNV infections. In this study, and for the first time, we demonstrate that NNV colonizes and replicates into the testis of gilthead seabream and European sea bass males, although its level of gene expression is very low and not easily detected by conventional or even real-time PCR methodologies. However, we localized the expression of the *cp* gene using *in situ* PCR as previously described on grouper embryos [18]. Thus, we localize the NNV *cp* RNA on Sertoli cells in the testis of both species, and on tunica albuginea’s and germ cells in the gilthead seabream testis. These results suggest that NNV could be spread into the germ cells of gilthead seabream and within the gonadal fluid of both species. However, as Sertoli cells are intimately associated to germ cells in fish [45], the shed of the virus into European sea bass germ cells cannot be discarded. Regarding viral proteins, we also immuno-detected the CP and B2 proteins in the Sertoli and/or interstitial cells of gilthead seabream testis. Interestingly, B2 protein production is only detected when the virus is performing an active infection instead of a persistent one [10]. In contrast to gilthead seabream, no proteins were detected in the testis of European sea bass; however we cannot discard very low rates of viral protein production in this specie. In fact, viral infected particles seems to be present in the testis of both species as we succeeded to recover the virus after one blind passage in a permissive cell line inoculated with testis homogenates from infected specimens of both species. Therefore, the lack of immunoreactivity with the anti-NNV sera could be due to the very low amount of NNV infective particles together with very low levels of gene transcription. Our data also exclude the possibility of blood contamination since the testis was rid of blood in most of the blood vessels, as observed in the histology samples, and the *in situ* PCR and immunocytochemistry techniques never showed viral mRNA or protein staining in blood vessels or cells (data not shown).

Other viruses have also demonstrated to colonize the testis, such as VHSV and IPNV in the rainbow trout and showed different replicating capacity and in turn they elicited a different immune response [26]. Interestingly, upon *in vivo* infection, IPNV did not trigger an effective immune response, which was triggered upon *in vitro* infection, so some extragonadal factors might block the immune response in the gonad improving the transmission of the virus [26]. NNV, however, triggered in the gilthead seabream testis a slight down-regulation of *tcrb* and *igmh* genes upon *in vivo* infection and of *il6* and *il1b* genes upon *in vitro* infection, whilst in the testis of European sea bass we observed up-regulations of the pro-inflammatory cytokine and *igmh* gene expressions upon *in vivo* infection and no changes upon *in vitro* infection. In fact, the same pattern in the testis expression of others immune-related genes, including antimicrobial peptides (AMPs) [47] and interferon (IFN) response [48], of both species was observed. Thus, the AMPs and IFN transcription is unaltered or inhibited in gilthead seabream testis, while in the European sea bass testis most of those genes are up-regulated upon *in vivo* and *in vitro* infections [47, 48]. Interestingly, in the gilthead seabream brain and head-kidney upon *in vivo* infection with NNV, the phagocytosis, the cell-mediated cytotoxic activity and the *mx* transcription *s*tarted very early upon the infection and stayed at high up to 15 days, while in the European sea bass brain these activities and the *mx* transcription was up-regulated at day 1 upon infection and quickly decreased to control levels [49]. Furthermore, our data showed that the pro-inflammatory cytokines are much more up-regulated in the European sea bass brain upon infection than in gilthead seabream. All these data, taken together, suggest that while gilthead seabream overcome the NNV infection and remove the virus from the brain probably due to a successful anti-viral immune response with little inflammatory consequences, the European sea bass fail to do so and the inflammatory response is up-regulated and probably produce high cell damage ([49] and this study data). Moreover, in the testis of gilthead seabream the immune response is tightly controlled and the virus succeeds to be transmitted as suggested by greater detection of NNV at gene, protein and infective particle levels. However, in the European sea bass, as the infection proceeds, the reproductive process become less important and the immune response try to keep the specimen alive, even when the tissue will be damaged, so the inflammatory response increases into the testis, together with the AMP and IFN responses, and could be the reason to the very low and limited detection of NNV. Interestingly, other studies described that NNV increased the number of cytotoxic T lymphocytes in the blood and up-regulates the expression of CD8α gene in groupers [50]. Similarly to what happened with the anti-viral immune response and the pro-inflammatory cytokines expression, the *tcrb* and *igmh* gene expression is up-regulated in the brain of gilthead seabream and down-regulated in the testis, while in the European sea bass only the *tcrb* is up-regulated in the brain upon 15 days of infection. Regarding the *igmh* transcription in European sea bass tissues, we observed a decrease at day 1 and an increase at day 15 in the brain, while in the testis this gene expression was up-regulated at day 7.

Several pathogen infections change the sex steroid hormone levels of the infected specimens [31, 32]. Curiously, low levels of E_2_ and high levels of testosterone (T) have been related with the progression of vibriosis symptoms [31]. Our data support this hypothesis as the E_2_ serum levels decreased as the infection proceeds only in the European sea bass, which showed mortality during the infection and displayed an earlier but less effective immune response in the brain [49]. In contrast, in the gilthead seabream, the E_2_ serum levels increased as the infection progressed. Moreover, in the last years, it has been described in the gilthead seabream that estrogens regulated the inflammatory immune response through endothelial cells and macrophage activation and increased leucocytes recruitment in the testis [51–54]. Furthermore, androgens such as testosterone also induced the recruitment of acidophilic granulocytes and IgM-positive cells in the testis of gilthead seabream and modulated *in vitro* the activity of gilthead seabream phagocytes and their sensitivity to pathogens [55, 56]. On the other hand, increases on E_2_ and T serum levels in the gilthead seabream, increased the complement and peroxidase activities at different time points although unmodified or decreased other humoral immune responses such as anti-protease activity and IgM serum levels [57]. Our data showed that NNV infection induced a strong increase on E_2_ serum levels at the beginning of the infection, whilst the serum levels of 11KT were decreased at these time points and increased at day 15. Taking into account that a certain increase in E_2_ serum levels stimulates the immune response in the gilthead seabream, this data also supports the ability of this species to fight against the NNV infection and overcome the disease.

In mammals, E_2_ regulates the inflammatory response as two-edges-sword, triggering stimulation or inhibition of this response depending on several parameters such as the immune stimulus, the tissue involved, the concentration of E_2_, the expression of estrogen receptors and so on [58]. Similarly, in fish, controversial data about the ability of sex steroid hormones to modulate the immune response has been published upon exogenous administration of several sex steroid hormones in several fish species [29, 57, 59]. We therefore studied the expression of some genes coding for some steroidogenic enzymes (*cyp11b1* and c*yp19a1a* and *cyp19a2*) and estrogen receptors (*era*, *erb1* and *erb2*) in the testis, the main organ that synthesizes biologically active steroids *de novo* [60], and brain, as the brain is the main target tissue of the virus, of both species upon *in vivo* infection and in the testis upon *in vitro* infection. Our data showed that in the gilthead seabream testis, the expression of the *cyp19a1a* gene that coded for the aromatase enzyme that transforms T to E_2_, was increased, coinciding with an increase on the E_2_ serum level. The expression of this gene was undetected in the European sea bass testis, and therefore the E_2_ serum levels decreased. However, the sensitivity of the gilthead seabream testis to E_2_ is decreased as the expression of the *era* at day 15 and of *erb1* and *erb2* at day 1 decreased after infection. On the other hand, we analysed the sensitivity of E_2_ and the local E_2_ production in the brain as locally produced E_2_ in the brain regulated important biological functions including reproduction and neuroprotection [61] and the brain is the main target tissue of the virus. Thus, we found that the expression of *era* in the brain increased at day 1 and decreased at day 7 upon infection whilst neither *erb1* nor *erb2* were detected. This is not surprising as, it has been suggested that the expression of *er* genes is not detected in some areas of the brain probably because its expression is very low [62]. In contrast to gilthead seabream brain, in European sea bass brain, the expression of the gene coding for the neural aromatase (*cyp19a2*), *erb1* and *erb2* were differently regulated. Regarding androgens, NNV decreases the 11KT serum levels of gilthead seabream without affecting, in the testis, the expression of the *cyp11b1* gene; that coded for the key enzyme in the transformation of T to 11KT in the testis. Although the testis is the main steroidogenic tissue, other tissues can synthesize 11KT or transform T to 11KT [63] producing the increase of serum 11KT observed upon NNV infection. Neither 11KT nor *cyp11b1* transcription has been detected in the brain of teleosts [62]. Regarding testicular functionality, the changes observed on sex steroid hormones levels and steroidogenic enzymes and estrogen receptor gene transcriptions seems not to be disrupted for the gilthead seabream testis as the GSI was increased and the *dmrt1* gene expression was slightly decreased upon 15 days of infection. However, the *dmrt1* transcription levels were not as low as needed to produce testicular disruption in gilthead seabream males [35]. Interestingly, the changes observed on the expression of all these genes in the testis of both species upon *in vitro* infection with NNV are different to those observed *in vivo* in most of the cases. This suggests that the changes in other tissues due to NNV infection; modifies the gonadal response upon infection.

## Conclusions

In conclusion, we have proved for the first time the ability of NNV to colonise the male testis of gilthead seabream and European sea bass and produce infective particles by means of *in situ* PCR, immunocytochemistry and cell culture. However, the response to the virus in both species is very different. In addition, NNV triggers the inflammatory immune response in the testis of European sea bass whilst seems to be overlooked in the gilthead seabream testis. This also applies to other immune responses, as AMPs or IFN production, previously studied, which could account for the higher presence of NNV in the seabream testis. Furthermore, we had also determined whether NNV is able to modulate the reproductive system to improve its transmission and could demonstrated that NNV alters E_2_ and 11KT production and the sensitivity of brain and testis to these hormones. Whether this is occurs due to changes in the fish physiological abilities to modulate the immune response or by NNV to improve its ability to replicate and be transmitted is still undetermined and further studies will be needed to understand these mechanisms. Apparently, according to the GSI and *dmrt1* expression levels, there is no disruption of testicular functions upon infection, which could favour the shedding and dissemination of the NNV to the water and/or the surrounding animals.

## Supporting Information

S1 File (Figure A) Transcription of the NNV capsid (*cp*) gene in European sea bass brain from control (C) and infected (I) fish at 15 days of *in vivo* infection with NNV. M, 100-bp ladder; lanes 1–2, control fish; lines 3–5, infected fish; line 6, negative control of the PCR; and line 7, purified NNV genome. (Figure B)The *in situ* PCR products do not diffuse to the aqueous phase. The liquid phase of each isPCR was run in a 2% agarose gel and showed no amplicons. M, 100-bp ladder; lanes 1–9 correspond to different samples and the bands to primers.
(TIF)

## References

[1] Munday BL, Kwang J, Moody N. Betanodavirus infections of teleost fish: a review. J Fish Dis. 2002;25:127–42.

[2] Gómez DK, Sato J, Mushiake K, Isshiki T, Okinaka Y, Nakai T. PCR-based detection of betanodaviruses from cultured and wild marine fish with no clinical signs. J Fish Dis. 2004;27(10):603–8. Epub 2004/10/16. doi: JFD577 [pii] 10.1111/j.1365-2761.2004.00577.x . 15482425

[3] Sommerset I, Nerland AH. Complete sequence of RNA1 and subgenomic RNA3 of Atlantic halibut nodavirus (AHNV). Dis Aquat Organ. 2004;58(2–3):117–25. Epub 2004/04/28. . 15109133 10.3354/dao058117

[4] Delsert C, Morin N, Comps M. A fish encephalitis virus that differs from other nodaviruses by its capsid protein processing. Arch Virol. 1997;142(12):2359–71. Epub 1997/01/01. . 9672600 10.1007/s007050050248

[5] Tan C, Huang B, Chang SF, Ngoh GH, Munday B, Chen SC, et al Determination of the complete nucleotide sequences of RNA1 and RNA2 from greasy grouper (*Epinephelus tauvina*) nervous necrosis virus, Singapore strain. J Gen Virol. 2001;82(Pt 3):647–53. Epub 2001/02/15. . 11172107 10.1099/0022-1317-82-3-647

[6] Chen NC, Yoshimura M, Guan HH, Wang TY, Misumi Y, Lin CC, et al Crystal Structures of a Piscine Betanodavirus: Mechanisms of Capsid Assembly and Viral Infection. PLoS Pathog. 2015;11(10):e1005203 Epub 2015/10/23. 10.1371/journal.ppat.1005203 PPATHOGENS-D-15-00772 [pii]. . 26491970 PMC4619592

[7] Iwamoto T, Mise K, Takeda A, Okinaka Y, Mori K, Arimoto M, et al Characterization of Striped jack nervous necrosis virus subgenomic RNA3 and biological activities of its encoded protein B2. J Gen Virol. 2005;86(Pt 10):2807–16. Epub 2005/09/28. doi: 86/10/2807 [pii] 10.1099/vir.0.80902-0 . 16186236

[8] Fenner BJ, Thiagarajan R, Chua HK, Kwang J. Betanodavirus B2 is an RNA interference antagonist that facilitates intracellular viral RNA accumulation. J Virol. 2006;80(1):85–94. 10.1128/jvi.80.1.85-94.2006 . 16352533 PMC1317529

[9] Nagai T, Nishzawa T. Sequence of the non-structural protein gene encoded by RNA1 of striped jack nervous necrosis virus. J Gen Virol. 1999;80(11):3019–22. 10580064 10.1099/0022-1317-80-11-3019

[10] Mezeth KB, Patel S, Henriksen H, Szilvay AM, Nerland AH. B2 protein from betanodavirus is expressed in recently infected but not in chronically infected fish. Dis Aquat Organ. 2009;83(2):97–103. Epub 2009/03/31. 10.3354/dao02015 . 19326790

[11] Breuil G, Bonami JR, Pepin JF, Pichot Y. Viral infection (picorna-like virus) associated with mass mortalities in hatchery-reared sea-bass (*Dicentrarchus labrax*) larvae and juveniles. Aquaculture. 1991;97(2–3):109–16. 10.1016/0044-8486(91)90258-9

[12] Frerichs G, Rodger HD, Peric Z. Cell culture isolation of piscine neuropathy nodavirus from juvenile sea bass, *Dicentrarchus labrax* . J Gen Virol. 1996;77:2067–71. 8811004 10.1099/0022-1317-77-9-2067

[13] Castric J, Thiery R, Jeffroy J, de Kinkelin P, Raymond JC. Sea bream *Sparus aurata*, an asymptomatic contagious fish host for nodavirus. Dis Aquat Organ. 2001;47(1):33–8. 10.3354/dao047033 . 11797913

[14] Olveira JG, Souto S, Dopazo CP, Thiery R, Barja JL, Bandin I. Comparative analysis of both genomic segments of betanodaviruses isolated from epizootic outbreaks in farmed fish species provides evidence for genetic reassortment. J Gen Virol. 2009;90(Pt 12):2940–51. Epub 2009/08/28. doi: vir.0.013912–0 [pii] 10.1099/vir.0.013912-0 . 19710256

[15] Breuil G, Pepin JFP, Boscher S, Thiery R. Experimental vertical transmission of nodavirus from broodfish to eggs and larvae of the sea bass, *Dicentrarchus labrax* (L.). J Fish Dis. 2002;25(12):697–702. 10.1046/j.1365-2761.2002.00406.x .

[16] Grotmol S, Bergh O, Totland GK. Transmission of viral encephalopathy and retinopathy (VER) to yolk-sac larvae of the Atlantic halibut *Hippoglossus hippoglossus*: occurrence of nodavirus in various organs and a possible route of infection. Dis Aquat Organ. 1999;36(0177–5103 (Print)):95–106. 10399037 10.3354/dao036095

[17] Korsnes K, Karlsbakk E, Nylund A, Nerland AH. Horizontal transmission of nervous necrosis virus between turbot *Scophthalmus maximus* and Atlantic cod *Gadus morhua* using cohabitation challenge. Dis Aquat Organ. 2012;99(1):13–21. 10.3354/dao02454 . 22585299

[18] Kuo HC, Wang TY, Hsu HH, Chen PP, Lee SH, Chen YM, et al Nervous Necrosis Virus Replicates Following the Embryo Development and Dual Infection with Iridovirus at Juvenile Stage in Grouper. PLoS ONE. 2012;7(4):e36183 10.1371/journal.pone.0036183 22563447 PMC3338570

[19] Sinyakov MS, Belotsky S, Shlapobersky M, Avtalion RR. Vertical and horizontal transmission of tilapia larvae encephalitis virus: The bad and the ugly. Virology. 2011;410(1):228–33. 10.1016/j.virol.2010.11.004 . 21131016

[20] Hodneland K, García R, Balbuena JA, Zarza C, Fouz B. Real-time RT-PCR detection of betanodavirus in naturally and experimentally infected fish from Spain. J Fish Dis. 2011;34(3):189–202. Epub 2011/02/11. 10.1111/j.1365-2761.2010.01227.x . 21306586

[21] Inoue R, Ushida K. Vertical and horizontal transmission of intestinal commensal bacteria in the rat model. FEMS Microbiol Ecol. 2003;46(2):213–9. 10.1016/S0168-6496(03)00215-0 19719575

[22] Chaves-Pozo E, Mulero V, Meseguer J, García-Ayala A. Professional phagocytic granulocytes of the bony fish gilthead seabream display functional adaptation to testicular microenvironment. J Leukoc Biol. 2005;78(2):345–51. Epub 2005/06/07. doi: jlb.0205120 [pii] 10.1189/jlb.0205120 . 15937143

[23] Hedger MP. Macrophages and the immune responsiveness of the testis. J Reprod Immunol. 2002;57(1–2):19–34. Epub 2002/10/19. doi: S0165037802000165 [pii]. . 12385831 10.1016/s0165-0378(02)00016-5

[24] Kocan RM, Hershberger PK, Elder NE. Survival of the North American strain of viral hemorrhagic septicemia virus (VHSV) in filtered seawater and seawater containing ovarian fluid, crude oil and serum-enriched culture medium. Dis Aquat Organ. 2001;44(1):75–8. Epub 2001/03/20. 10.3354/dao044075 . 11253879

[25] Smail DA, Munro ES. Isolation and quantification of infectious pancreatic necrosis virus from ovarian and seminal fluids of Atlantic salmon, *Salmo salar* L. J Fish Dis. 2008;31(1):49–58. Epub 2007/12/19. doi: JFD866 [pii] 10.1111/j.1365-2761.2007.00866.x . 18086034

[26] Chaves-Pozo E, Montero J, Cuesta A, Tafalla C. Viral hemorrhagic septicemia and infectious pancreatic necrosis viruses replicate differently in rainbow trout gonad and induce different chemokine transcription profiles. Dev Comp Immunol. 2010;34(6):648–58. Epub 2010/01/26. doi: S0145-305X(10)00022-4 [pii] 10.1016/j.dci.2010.01.009 . 20096727

[27] Migaud H, Ismail R, Cowan M, Davie A. Kisspeptin and seasonal control of reproduction in male European sea bass (*Dicentrarchus labrax*). Gen Comp Endocrinol. 2012;179(3):384–99. Epub 2012/10/06. doi: S0016-6480(12)00381-4 [pii] 10.1016/j.ygcen.2012.07.033 . 23036731

[28] Weltzien FA, Andersson E, Andersen O, Shalchian-Tabrizi K, Norberg B. The brain-pituitary-gonad axis in male teleosts, with special emphasis on flatfish (Pleuronectiformes). Comp Biochem Physiol A Mol Integr Physiol. 2004;137(3):447–77. Epub 2004/05/05. 10.1016/j.cbpb.2003.11.007 S1095643303003568 [pii]. . 15123185

[29] Chaves-Pozo E, Cabas I, García-Ayala A. Sex steroids modulate fish immune response In: Kahn SM, editor. Sex steroids. Rijeka: InTech; 2012 p. 199–220.

[30] Muñoz-Cruz S, Togno-Pierce C, Morales-Montor J. Non-reproductive effects of sex steroids: their immunoregulatory role. Curr Top Med Chem. 2011;11(13):1714–27. Epub 2011/04/06. doi: BSP/CTMC/E-Pub/-000100-11-12 [pii]. . 21463251 10.2174/156802611796117630

[31] Deane EE, Li J, Woo NY. Hormonal status and phagocytic activity in sea bream infected with vibriosis. Comp Biochem Physiol B Biochem Mol Biol. 2001;129(2–3):687–93. Epub 2001/06/12. doi: S1096495901003694 [pii]. . 11399506 10.1016/s1096-4959(01)00369-4

[32] Gómez Y, Valdez RA, Larralde C, Romano MC. Sex steroids and parasitism: *Taenia crassiceps cisticercus* metabolizes exogenous androstenedione to testosterone in vitro. J Steroid Biochem Mol Biol. 2000;74(3):143–7. Epub 2000/11/22. doi: S0960-0760(00)00099-6 [pii]. . 11086233 10.1016/s0960-0760(00)00099-6

[33] Blázquez M, Navarro-Martín L, Piferrer F. Expression profiles of sex differentiation-related genes during ontogenesis in the European sea bass acclimated to two different temperatures. J Exp Zool B Mol Dev Evol. 2009;312(7):686–700. Epub 2009/04/02. 10.1002/jez.b.21286 . 19338052

[34] Reed LJ, Müench A. A simple method of stimating fifty percent end points. Am J Hyg. 1938;27:493–7.

[35] Aranguren R, Tafalla C, Novoa B, Figueras A. Experimental transmission of encephalopathy and retinopathy induced by nodavirus to sea bream, *Sparus aurata* L., using different infection models. J Fish Dis. 2002;25(6):317–24. 10.1046/j.1365-2761.2002.00368.x .

[36] Nuovo GJ. In situ PCR: protocols and applications. PCR Methods Appl. 1995;4(4):151–67. Epub 1995/02/01. . 8574184 10.1101/gr.4.4.s151

[37] Iwamoto T, Nakai T, Mori K, Arimoto M, Furusawa I. Cloning of the fish cell line SSN-1 for piscine nodaviruses. Dis Aquat Organ. 2000;43(2):81–9. Epub 2001/01/06. 10.3354/dao043081 . 11145456

[38] Nishizawa T, Mori K, Nakai T, Furuzawa I, Muroga K. Polymerase chain reaction (PCR) amplification of RNA of striped jack nervous necrosis virus (SJNNV). Dis Aquat Organ. 1994;18:103–7.

[39] Olveira JG, Souto S, Dopazo CP, Bandín I. Isolation of betanodavirus from farmed turbot *Psetta maxima* showing no signs of viral encephalopathy and retinopathy. Aquaculture. 2013;406â€“407(0):125–30. 10.1016/j.aquaculture.2013.05.007

[40] Rodríguez L, Begtashi I, Zanuy S, Carrillo M. Development and validation of an enzyme immunoassay for testosterone: Effects of photoperiod on plasma testosterone levels and gonadal development in male sea bass (*Dicentrarchus labrax*, L.) at puberty. Fish Physiol Biochem. 2000;23(2):141–50. 10.1023/a:1007871604795

[41] Chaves-Pozo E, Arjona FJ, García-López A, García-Alcázar A, Meseguer J, García-Ayala A. Sex steroids and metabolic parameter levels in a seasonal breeding fish (*Sparus aurata* L.). General and comparative endocrinology. 2008;156(3):531–6. Epub 2008/04/15. doi: S0016-6480(08)00114-7 [pii] 10.1016/j.ygcen.2008.03.004 . 18407272

[42] Valero Y, Sánchez-Hernández M, García-Alcázar A, García-Ayala A, Cuesta A, Chaves-Pozo E. Characterization of the annual regulation of reproductive and immune parameters on the testis of European sea bass. Cell Tissue Res. 2015 Epub 2015/04/22. 10.1007/s00441-015-2172-1 . 25896883

[43] Chaves-Pozo E, Mulero V, Meseguer J, García-Ayala A. An overview of cell renewal in the testis throughout the reproductive cycle of a seasonal breeding teleost, the gilthead seabream (*Sparus aurata* L.). Biol Reprod. 2005;72(3):593–601. 10.1095/biolreprod.104.036103 . 15548730

[44] Liarte S, Chaves-Pozo E, García-Alcázar A, Mulero V, Meseguer J, García-Ayala A. Testicular involution prior to sex change in gilthead seabream is characterized by a decrease in DMRT1 gene expression and by massive leukocyte infiltration. Reprod Biol Endocrinol. 2007;5:20–35. doi: 20 10.1186/1477-7827-5-20 . 17547755 PMC1894798

[45] Schulz RW, de Franca LR, Lareyre JJ, Le Gac F, Chiarini-Garcia H, Nobrega RH, et al Spermatogenesis in fish. Gen Comp Endocrinol. 2010;165(3):390–411. Epub 2009/04/08. doi: S0016-6480(09)00074-4 [pii] 10.1016/j.ygcen.2009.02.013 . 19348807

[46] Maltese C, Bovo G. Viral encephalopathy and retitopathy. Ittiopatologia 2007;4:93–146.

[47] Valero Y, García-Alcazar A, Esteban MA, Cuesta A, Chaves-Pozo E. Antimicrobial response is increased in the testis of European sea bass, but not in gilthead seabream, upon nodavirus infection. Fish Shellfish Immunol. 2015;44:203–13. Epub 2015/02/25. doi: S1050-4648(15)00068-6 [pii] 10.1016/j.fsi.2015.02.015 . 25707600

[48] Valero Y, Morcillo P, Meseguer J, Buonocore F, Esteban MA, Chaves-Pozo E, et al Characterization of the interferon pathway in the teleost fish gonad against the vertically transmitted viral nervous necrosis virus. J Gen Virol. 2015. Epub 2015/04/29. doi: vir.0.000164 [pii] 10.1099/vir.0.000164 . 25918238

[49] Chaves-Pozo E, Guardiola FA, Meseguer J, Esteban MA, Cuesta A. Nodavirus infection induces a great innate cell-mediated cytotoxic activity in resistant, gilthead seabream, and susceptible, European sea bass, teleost fish. Fish Shellfish Immunol. 2012;33(5):1159–66. Epub 2012/09/18. doi: S1050-4648(12)00314-2 [pii] 10.1016/j.fsi.2012.09.002 . 22981914

[50] Chang YT, Kai YH, Chi SC, Song YL. Cytotoxic CD8alpha+ leucocytes have heterogeneous features in antigen recognition and class I MHC restriction in grouper. Fish Shellfish Immunol. 2011;30(6):1283–93. Epub 2011/04/06. doi: S1050-4648(11)00122-7 [pii] 10.1016/j.fsi.2011.03.018 . 21463694

[51] Cabas I, Chaves-Pozo E, García-Alcazar A, Meseguer J, Mulero V, García-Ayala A. Dietary intake of 17alpha-ethinylestradiol promotes leukocytes infiltration in the gonad of the hermaphrodite gilthead seabream. Mol Immunol. 2011;48(15–16):2079–86. Epub 2011/08/09. 10.1016/j.molimm.2011.07.001 . 21821292

[52] Chaves-Pozo E, Liarte S, Vargas-Chacoff L, García-López A, Mulero V, Meseguer J, et al 17Beta-estradiol triggers postspawning in spermatogenically active gilthead seabream (*Sparus aurata* L.) males. Biol Reprod. 2007;76(1):142–8. Epub 2006/10/20. 10.1095/biolreprod.106.056036 . 17050857

[53] Liarte S, Chaves-Pozo E, Abellán E, Meseguer J, Mulero V, Canario AV, et al Estrogen-responsive genes in macrophages of the bony fish gilthead seabream: A transcriptomic approach. Dev Comp Immunol. 2011;35:840–9. Epub 2011/03/23. 10.1016/j.dci.2011.03.015 . 21420425

[54] Liarte S, Chaves-Pozo E, Abellán E, Meseguer J, Mulero V, García-Ayala A. 17beta-Estradiol regulates gilthead seabream professional phagocyte responses through macrophage activation. Dev Comp Immunol. 2011;35:19–27. Epub 2010/08/10. 10.1016/j.dci.2010.07.007 . 20692288

[55] Águila S, Castillo-Briceño P, Sánchez M, Cabas I, García-Alcázar A, Meseguer J, et al Specific and non-overlapping functions of testosterone and 11-ketotestosterone in the regulation of professional phagocyte responses in the teleost fish gilthead seabream. Mol Immunol. 2012;53(3):218–26. Epub 2012/09/11. doi: S0161-5890(12)00361-6 [pii] 10.1016/j.molimm.2012.08.002 . 22960553

[56] Sánchez-Hernández M, Chaves-Pozo E, Cabas I, Mulero V, García-Ayala A, García-Alcázar A. Testosterone implants modify the steroid hormone balance and the gonadal physiology of gilthead seabream (*Sparus aurata* L.) males. J Steroid Biochem Mol Biol. 2013;138C:183–94. Epub 2013/06/08. 10.1016/j.jsbmb.2013.05.014 . 23743364

[57] Cuesta A, Vargas-Chacoff L, García-López A, Arjona FJ, Martínez-Rodríguez G, Meseguer J, et al Effect of sex-steroid hormones, testosterone and estradiol, on humoral immune parameters of gilthead seabream. Fish Shellfish Immunol. 2007;23(3):693–700. 10.1016/j.fsi.2007.01.015 17349804

[58] Straub RH. The complex role of estrogens in inflammation. Endocr Rev. 2007;28(5):521–74. Epub 2007/07/21. doi: er.2007-0001 [pii] 10.1210/er.2007-0001 . 17640948

[59] Hou Y, Suzuki Y, Aida K. Effects of steroids on the antibody producing activity of lymphocytes in rainbow trout. Fisheries science. 1999;65(6):850–5. 10.2331/fishsci.65.850

[60] Stocco DM. StAR protein and the regulation of steroid hormone biosynthesis. Annu Rev Physiol. 2001;63:193–213. Epub 2001/02/22. 10.1146/annurev.physiol.63.1.193 63/1/193 [pii]. . 11181954

[61] García-Segura LM, Azcoitia I, DonCarlos LL. Neuroprotection by estradiol. Prog Neurobiol. 2001;63(1):29–60. Epub 2000/10/21. doi: S0301-0082(00)00025-3 [pii]. . 11040417 10.1016/s0301-0082(00)00025-3

[62] Diotel N, Do Rego JL, Anglade I, Vaillant C, Pellegrini E, Vaudry H, et al The brain of teleost fish, a source, and a target of sexual steroids. Front Neurosci. 2011;5:137–53. Epub 2011/12/24. 10.3389/fnins.2011.00137 . 22194715 PMC3242406

[63] Arukwe A. Steroidogenic acute regulatory (StAR) protein and cholesterol side-chain cleavage (P450scc)-regulated steroidogenesis as an organ-specific molecular and cellular target for endocrine disrupting chemicals in fish. Cell Biol Toxicol. 2008;24(6):527–40. Epub 2008/04/10. 10.1007/s10565-008-9069-7 . 18398688

